# Supplementary material for: Sopa: a technology-invariant pipeline for analyses of image-based spatial omics
Source: Nat Commun. 2024 Jun 11;15:4981. doi: 10.1038/s41467-024-48981-z (PMC11167053; doi:10.1038/s41467-024-48981-z)
Supplement: Supplementary file 1 — Supplementary Information [file 41467_2024_48981_MOESM1_ESM.pdf]

Supplementary Information

Sopa: a technology-invariant pipeline for analyses of image-based spatial omics

Quentin Blampey[1,2*#], Kevin Mulder[3#], Margaux Gardet[3], Stergios Christodoulidis[1], Charles-Antoine Dutertre[3], Fabrice André[2,4], Florent Ginhoux[3] & Paul-Henry Cournède[1*]

[1] *Paris-Saclay University, CentraleSupélec, Laboratory of Mathematics and Computer Science (MICS), Gif-sur-Yvette, France*

[2] *Paris-Saclay University, Gustave Roussy, INSERM U981, Villejuif, France*

[3] *Paris-Saclay University, Gustave Roussy, INSERM U1015, Villejuif, France*

[4] *Gustave Roussy, Department of Medical Oncology, Villejuif, France*

# These authors contributed equally

*Correspondence to: quentin.blampey@gmail.com (Quentin Blampey)

*Correspondence to: paul-henry.cournede@centralesupelec.fr (Paul-Henry Cournède)

Supplementary Notes

Choice of SpatialData as a data structure

SpatialData[1] is a data structure developed in Python that aims to store spatial-related objects. It also provides transformations between coordinate systems (for instance, between microns and pixels), lazy representation for large images with Dask[2] and Xarray[3], transcripts stored as Dask[2] dataframes, and cells polygons stored as GeoPandas[4] polygons. The general structure of this data, the community support, and integration with the scverse[5] ecosystem make it a reliable tool to store spatial omics objects in Sopa. Notably, the usage of Python is appreciated since most recent models in spatial omics are gradually moving to Python for package development[6].

Integration with the scverse ecosystem

The scverse[5] ecosystem is a Python-based suite of fundamental tools for single-cell omics data analysis. This includes the data structures SpatialData[1] that we use for Sopa, as well as Scanpy[7], which covers a wide range of use cases in single-cell analysis. Also, still in the scverse ecosystem, Squidpy[8] is a Python library for the analysis of spatial single-cell data such as spatial neighbourhood analysis or ligand-receptor interaction analysis. Since Squidpy supports SpatialData, Sopa also naturally integrates with Squidpy. Indeed, the pipeline output is a SpatialData object, and Squidpy can operate on this, enabling all Squidpy functionalities to be leveraged after Sopa or inside the pipeline. Squidpy is complementary to Sopa since it operates on processed spatial omics, contrary to Sopa, which analyses raw data. Also, the spatial statistics tools available in Sopa do not exist in Squidpy. Thus, these packages have non-overlapping and complementary functionalities.

Limitation of the proprietary visualization software

All visualizers are exclusive to their data structure and require an investment of time from the users to learn their proprietary software. Besides this, some of the software comes only with the purchased machine and requires a license key for use. This limits the number of users who have a collaborative engagement and are not in possession of the machine. Data analysis from the MERSCOPE comes with a dedicated visualizer, called the ”Merscope Visualizer”. Its input is proprietary *”.vzg”* files, a non-open format. While VPT offers the possibility to update it, a new *vzg* cannot be recreated for another type of technology. In addition, the update of this file requires performing again all required operations, even for minor changes, because everything is included in one file. Therefore, minor modifications still imply a significant runtime to be updated in the visualizer. Concerning CosMX data, they offer an online suite of tools, called AtoMx, which is cloud-based only, limiting the accessibility, especially for users wanting to use their own high-

processing-cluster. Concerning the visualizer of the PhenoCycler and MACSima, they are specific to multiplex imaging, i.e. no transcript can be shown. Contrary to the other visualizers, Xenium Explorer can be both (i) downloaded freely and (ii) supports open file formats. This makes it a reliable choice for conversion from SpatialData. Also, it supports missing data, i.e. it will not crash when reading multiplex imaging data (from which no transcripts are available).

Visualization with the Xenium Explorer

After using Sopa, the files required by the Xenium Explorer are created. In particular, a file called "experiment.xenium" can be opened in the Xenium Explorer. The later software is freely available for both Windows and MacOS. Sopa has been tested on versions 1.2 and 1.3 of the Xenium Explorer. We show two examples of visualization in Supplementary Fig. 2 (Xenium dataset, 10X) and Supplementary Fig. 3 (MERSCOPE dataset, Vizgen).

Image alignment with the Xenium Explorer

One challenge for spatial transcriptomics can be to align images from different technologies when they are run on the same sample. Most of the time, a simple affine transformation is enough to align them. Since Sopa create outputs in the Xenium Explorer, it is possible to use the alignment tool available on the software. It consists of applying some mirroring transformations, rotations, and alignment based on user-defined reference points. Then, the transformation matrix can be saved via the visualizer, which will create a "matrix.csv" transformation file. Afterwards, we can use this transformation matrix to align the new image on our SpatialData object and perform any operation available in Sopa. This can be done via the Sopa CLI, by specifying `sopa explorer add-aligned <sdata_path> <image_path> <matrix_path>`. Typically, when adding an IF image, we can compute the mean channel intensity for all cells and for all channels.

Thresholds for conflict resolution

When two cell boundaries are overlapping, we compute their intersection-over-min-area (IOMA) to determine whether or not to merge the cell boundaries. In this section, we define thresholds of IOMA scores to be considered as good conflict resolution in Figure c/d/e. The upper bound is defined based on the value such that two cells randomly overlapping have a 0.025 probability of having an IOMA higher than this upper bound. Specifically, let $R \in \mathbb{R}^+$ a cell radius and two cells of radius R whose center are C_1 and $C_2 \in \mathbb{R}^2$, respectively. Without loss of generability, we consider that C_1 is centered in the 2D plane, that is $C_1 = (0, 0)$. Since the two cells are overlapping, we assume that C_2 is a random variable uniformly distributed on the circle of radius $2R$, that is $C_2 \sim \mathcal{U}(\{(x, y) \in \mathbb{R}^2, \sqrt{x^2 + y^2} \leq 2R\})$. Now, let D the random variable representing the distance between the two cells, i.e. $D = ||C_1||_2$. Finally, the quantile Q_p is defined as by the equation $P(f(D) \leq Q_p) = p$, where $p \in [0, 1]$ and f is the function that computes the IOMA score, that is, $f(D) = \frac{2}{\pi}\left(\arccos(\frac{D}{2R}) - \frac{D}{2R}\sqrt{1 - \frac{D^2}{4R^2}}\right)$. Since f decreases with respect to D, we have $P(f(D) \leq Q_p) = P(D \geq f^{-1}(Q_p)) = 1 - \frac{f^{-1}(Q_p)^2}{4R^2}$. This leads to $Q_p = \frac{2}{\pi}\left(\arccos(\sqrt{1-p}) - \sqrt{p(1-p)}\right)$, and, in particular, $Q_{0.975} \approx 0.7995$. Concerning the lower bound, the computation is different, because of the nature of such overlaps. Indeed, since the images have a certain thickness, two non-touching cells can appear as overlapping when projected on a 2D plane. We want to compute the mean IOMA of two non-touching cells that are overlapping when projected on the (x,y) plane. For that, we define L, the thickness of a slide. Let $Z_1, Z_2 \sim \mathcal{U}([-\frac{L}{2}, \frac{L}{2}])$ the random variables representing the position of the center of two cells on the z-axis. Again, we suppose that one cell is centered on $X_1 = 0$, while X_2 is assumed to be uniformly distributed while following these two conditions: (i) $X_2 \leq 2R$, in order for the two cells to overlap on the (x,y) plane, and (ii) $X_2^2 + (Z_1 - Z_2)^2 \geq 4R^2$ so that the two cells are not touching each other on the (x,y,z) space. Note that, when projected on the (x,y) plane, the distance between the

two cells is X_2. Computational simulations gives $\mathbb{E}(f(X_2)) = 0.07$, which is used as our lower bound (see supplementary Supplementary Fig. 8 for the full distribution).

Synthetic dataset generation

In order to demonstrate Sopa's efficiency on multiple dataset sizes, we created synthetic datasets. Let L be the width of the image, and d be the cell density in the image. An evenly distributed grid of size $(L\sqrt{d}, L\sqrt{d})$ is generated, each vertex corresponding to a cell location. We apply a Gaussian noise of standard-deviation $\frac{1}{2\sqrt{d}}$ on these cell locations to have a more natural distribution of cells. Images are generated by applying a Gaussian blur of standard deviation $\frac{1}{2\sqrt{d}}$ on the pixels at the location of the cell vertices, and 100 transcripts per cell are generated via a 2D Gaussian distribution of the same standard deviation.

Annotation of example datasets

Dataset annotation followed the procedure outlined in the main manuscript. Automatic annotation utilized the following references: Liver dataset (`https://www.immunesinglecell.org/atlas/liver`) and Pancreas dataset (`https://www.immunesinglecell.org/atlas/pancreas`). Initial global annotation involved combining major cell populations, followed by refinement using Leiden clustering[9]. Subsequent in-depth analysis employed manual annotation with Leiden clustering. For MACSima and PhenoCycler datasets, exclusion criteria involved DAPI, boundary staining, and low-quality proteins to enhance resolution. Manual clustering with Leiden was then applied for population annotation. Niche calculations were performed using STAGATE[10]. Niches were annotated based on cell type abundance and tissue structure, validated by a pathologist.

Comments on Baysor performances on patches

On Figure 3c/f, Baysor[11] had a better DE score when running on the patches than without. Actually, running Baysor on patches simplifies the complexity of each run since it will focus on a subset of cell types, which could increase Baysor specificity. For instance, for a patch that is specific to the stroma, Baysor may have an enhanced resolution compared to a run on the full image (which contains a broader range of cell types). This may explain why it has more power for DE, and it is opening up potential investigations beyond this paper to confirm this explanation.

Supplementary Tables

	MERSCOPE dataset	Xenium dataset	Phenocycler dataset	MACSima dataset
Matching cells without merge	11960 (98.1%)	69063 (97.5%)	892845 (99.5%)	42230 (99.1%)
Matching cells with merge	227 (1.86%)	1738 (2.45%)	4753 (0.529%)	381 (0.894%)
Non-matching cells	1 (0.0082%)	7 (0.00989%)	54 (0.00602%)	4 (0.00939%)

Supplementary Table 1: **Detailed status of cells during conflict resolution for the crops of size (16000x16000) of the original image.** Each resulting cell is separated into three categories. First, cells for which no conflict was found. Secondly, cells for which conflict was resolved but whose resulting cell corresponds to one unique cell in the segmentation without patches. Third, cells for which conflict was resolved but does not correspond to one unique cell in the segmentation without patches. Both raw numbers and percentages are provided.

Supplementary Figures

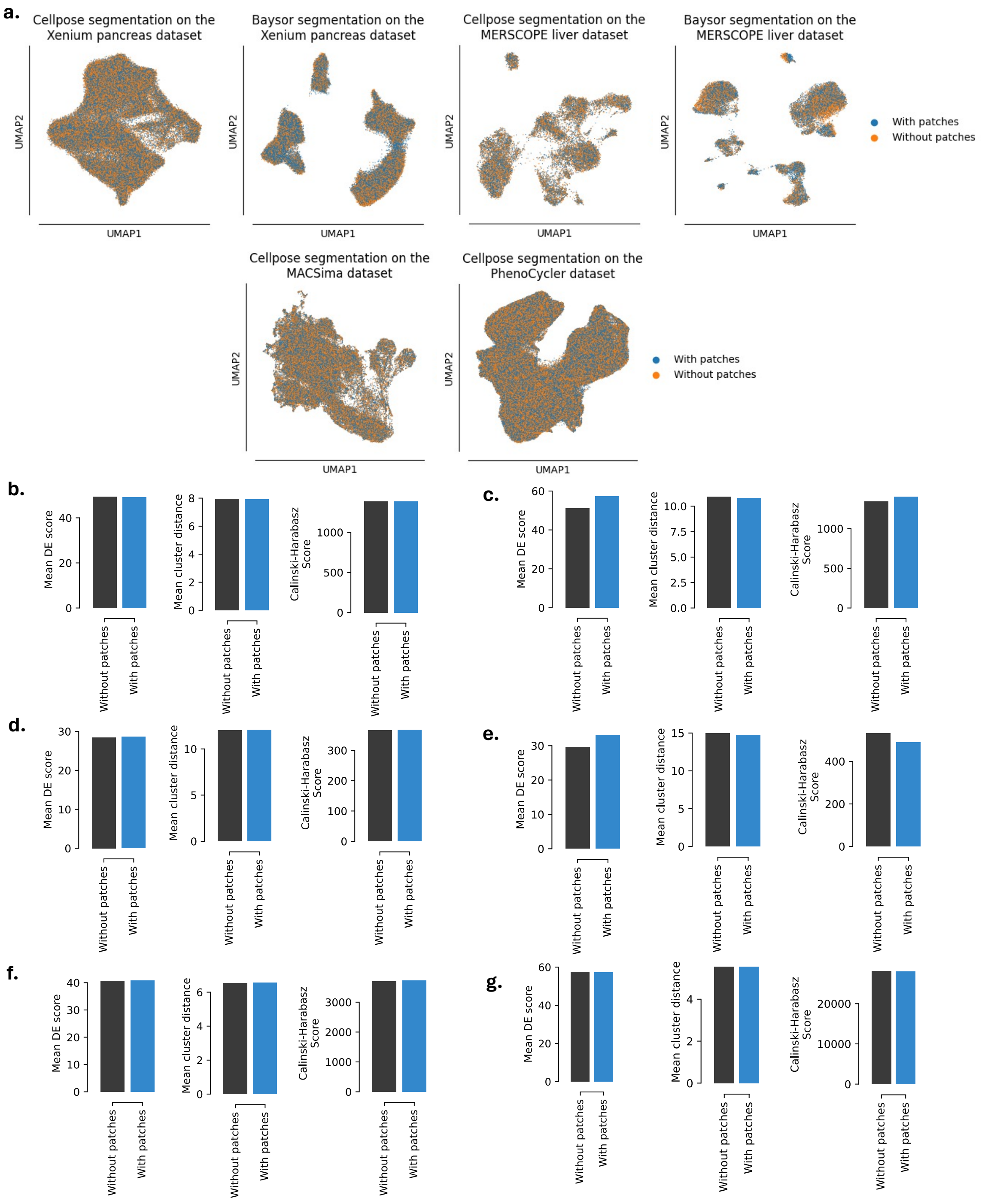

Supplementary Figure 1: **Impact of patched-based segmentation over the data quality, based on crops of size (16000x16000) of the original image. a.** UMAPs comparing the representation of the cells obtained while running segmentation over the whole image and using patches (as in Sopa). This was tested over multiple datasets, for Cellpose (for all datasets) and Baysor (for spatial transcriptomics datasets). **b.** Comparison of segmentation quality metrics for Cellpose run on the Xenium dataset. **c.** Comparison of segmentation quality metrics for Baysor run on the Xenium dataset. **d.** Comparison of segmentation quality metrics for Cellpose run on the MERSCOPE dataset. **e.** Comparison of segmentation quality metrics for Baysor run on the MERSCOPE dataset. **f.** Comparison of segmentation quality metrics for Cellpose run on the MACSima dataset. **g.** Comparison of segmentation quality metrics for Cellpose run on the PhenoCycler dataset.

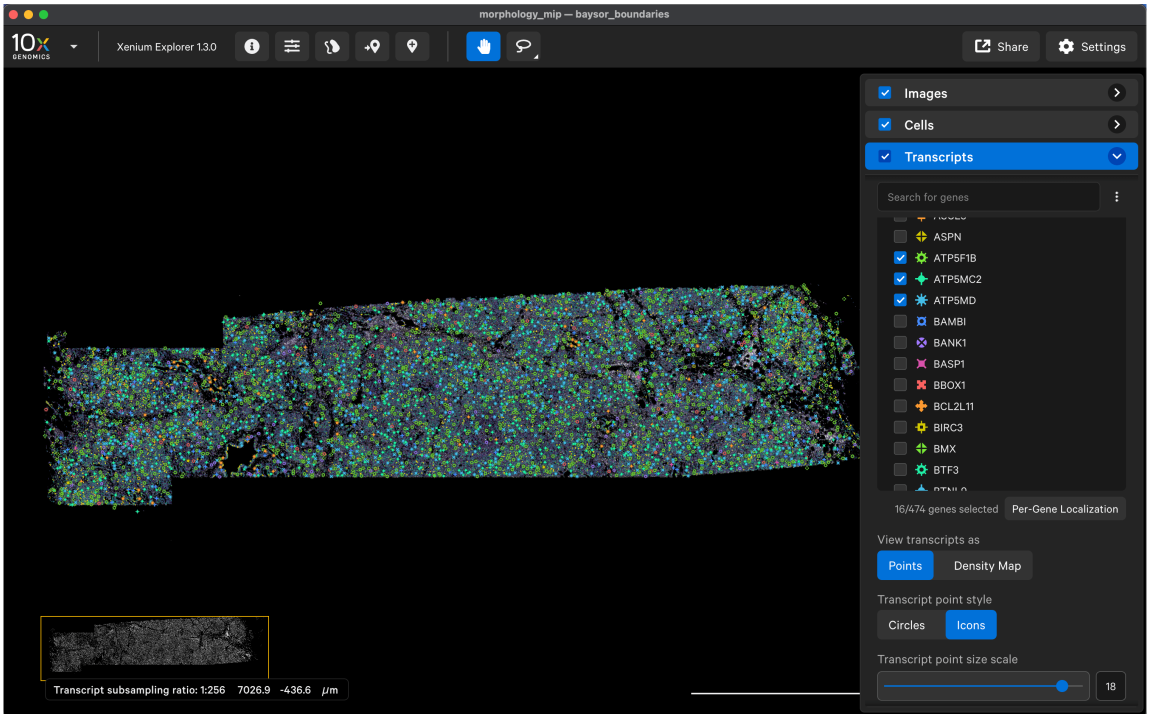

Supplementary Figure 2: Xenium human pancreatic cancer dataset (10X Genomics) open in the Xenium Explorer. The transcript panel is shown, with a few genes selected. Cells are coloured by a colour gradient representing transcript count.

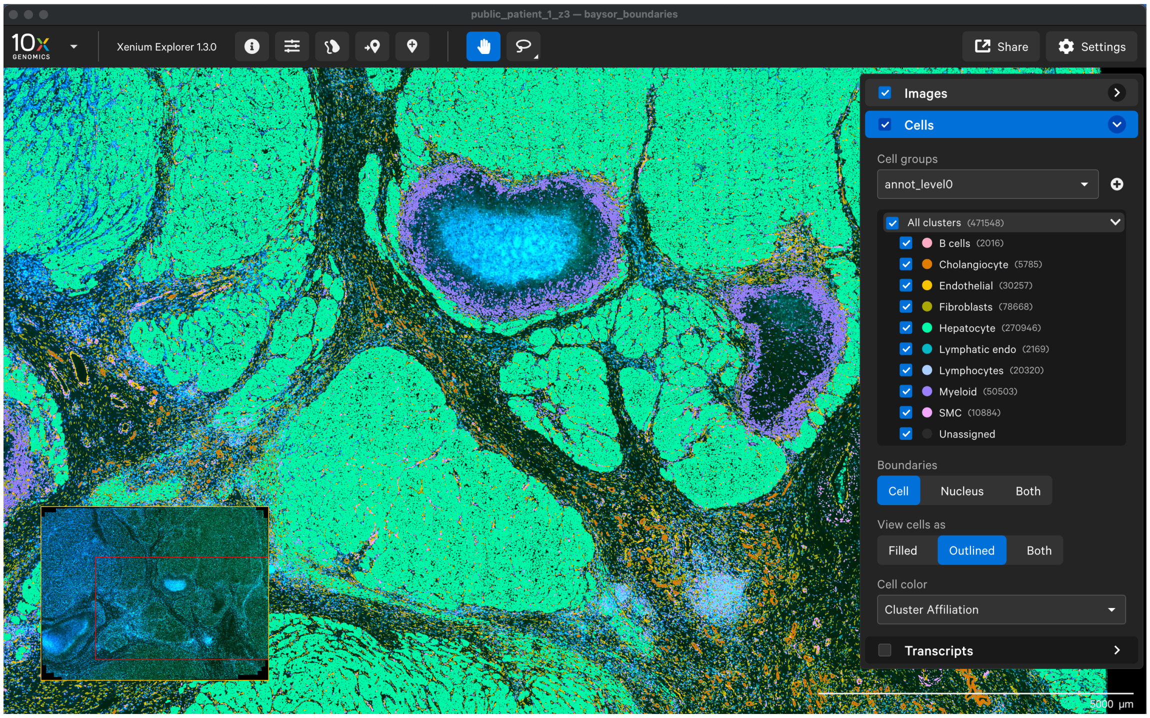

Supplementary Figure 3: MERSCOPE human liver hepatocellular carcinoma dataset (Vizgen) open in the Xenium Explorer. The cell panel is shown, and the "*annot_level0*" category is displayed. Colors correspond to a cell-type.

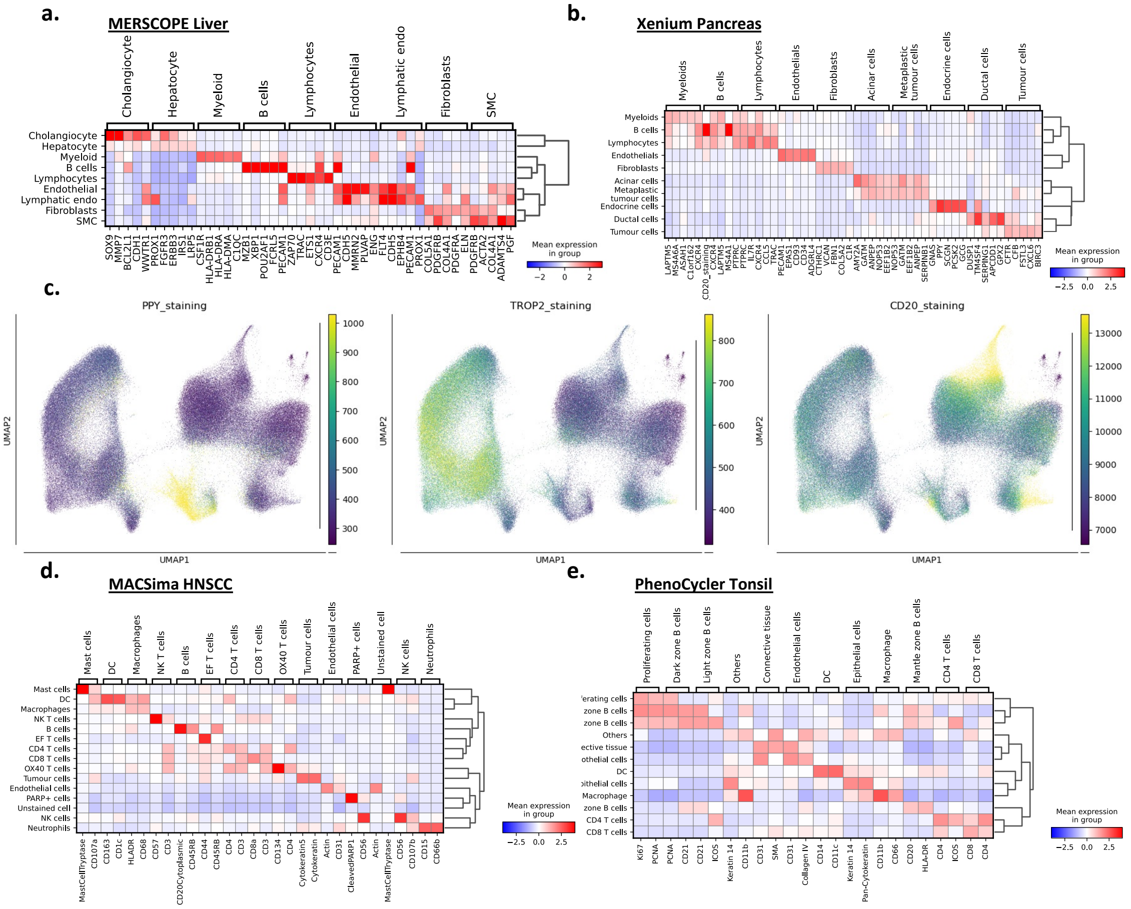

Supplementary Figure 4: **Visual validation of the annotations. a.** Heatmap of genes expression per population on the MERSCOPE human liver hepatocellular carcinoma dataset. **b.** Heatmap of genes expression per population on the Xenium pancreas dataset. **c.** Protein staining per cell on the Xenium human pancreatic cancer dataset after aligning the staining image to the original Xenium image. **d.** Heatmap of protein expression per population on the MACSima HNSCC dataset. **e.** Heatmap of protein expression per population on the Phenocycler tonsil dataset.

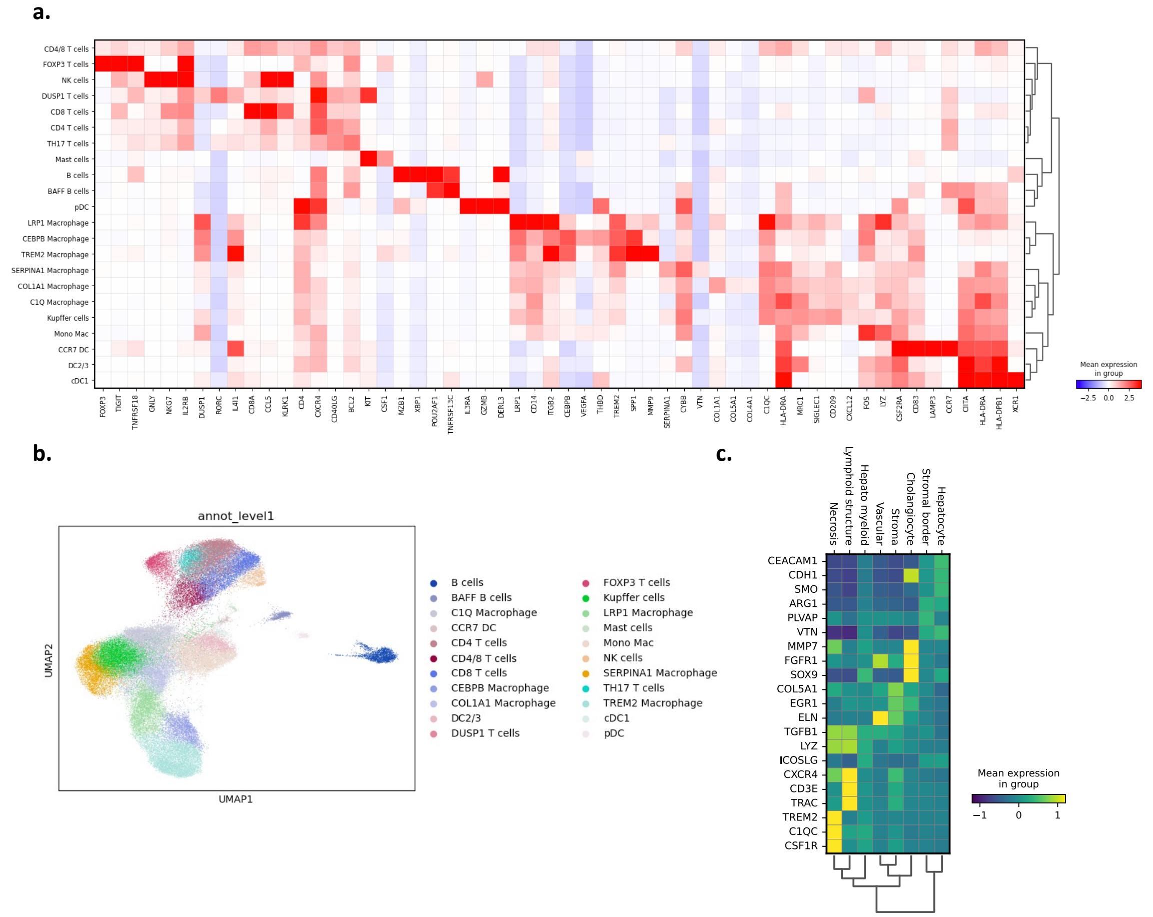

Supplementary Figure 5: **Annotation of the immune cells of the MERSCOPE human liver hepatocellular carcinoma dataset and niche differential gene expressions (DEGs). a.** Heatmap of DEGs per immune population on the MERSCOPE liver dataset. **b.** UMAP of immune cells of the MERSCOPE liver dataset **c.** Heatmap of DEGs per niche of the MERSCOPE liver dataset.

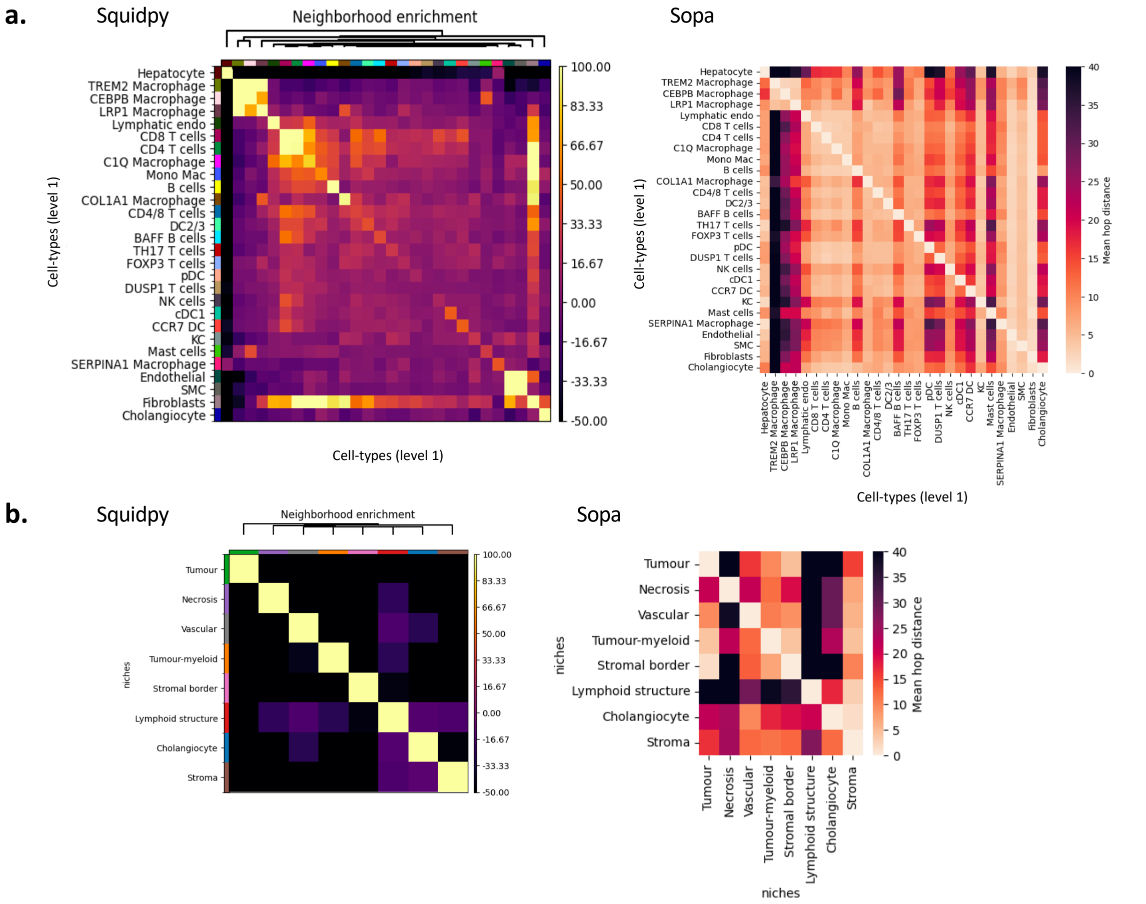

Supplementary Figure 6: **Comparison between Squidpy and Sopa post-processing analyses a.** Comparison of Squidpy neighbourhood enrichment (cell to cell) and Sopa cell to cell average hop distance. While the neighbourhood enrichment is symmetric, the distances are not. In terms of insights, this asymmetry can, for instance, show that *TREM2* macrophages are relatively close to the Hepatocytes, while the Hepatocytes are generally far from the *TREM2* Macrophages. To prevent confusion while reading this heatmap, we precise that one row corresponds to the distances from the cell type of the row index to all other cell types. **b.** Comparison of Squidpy neighbourhood enrichment (niche to niche) and Sopa niche to niche average hop distance. Since niches are more global structures, their neighbourhood usually includes only the same niche (left), while the distances can capture more global organizations and information (right).

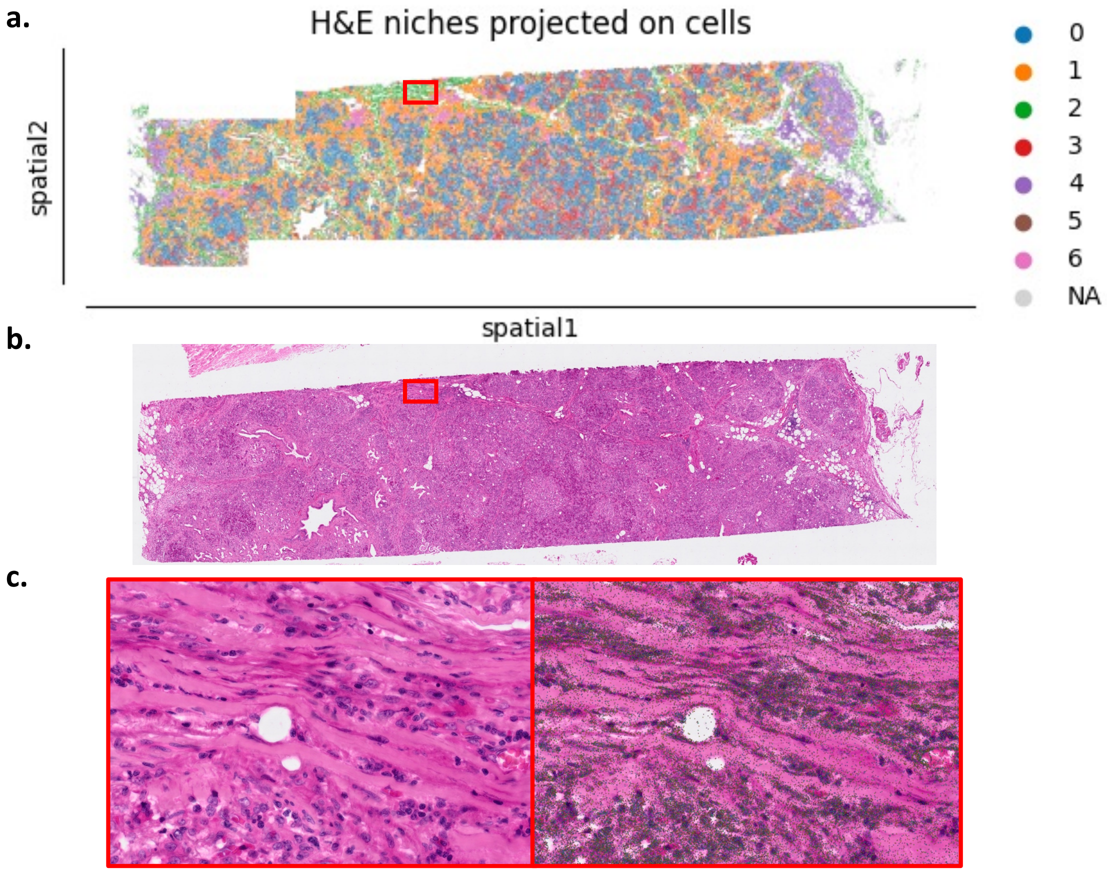

Supplementary Figure 7: **Zoom on Xenium human pancreatic cancer dataset a.** H&E clusters of patch-level embeddings based on a pre-trained computer vision model (denoted as H&E niches), red box highlighting the image in c. **b.** H&E image of human pancreatic cancer dataset (10X Genomics dataset), red box highlighting the image in c. **c.** Left, zoom of H&E image of human pancreatic cancer dataset (10X Genomics dataset). Right, zoom of H&E image with all transcript overlay of human pancreatic cancer dataset (10X Genomics dataset).

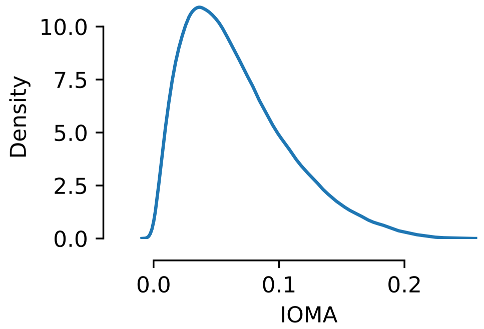

Supplementary Figure 8: **Distribution of IOMA when non-touching 3D cells are projected on a 2D plane.** This computation has been performed by simulating random non-touching 3D cells.

Supplementary References

1. Marconato, L. et al. SpatialData: an open and universal data framework for spatial omics. Nat Methods 1–5 (2024) doi:10.1038/s41592-024-02212-x.

2. Dask Development Team (2016). Dask: Library for dynamic task scheduling.

3. Hoyer, S. & Hamman, J. xarray: N-D labeled Arrays and Datasets in Python. 5, 10 (2017).

4. geopandas/geopandas: v0.8.1. doi:10.5281/zenodo.3946761.

5. Virshup, I. et al. The scverse project provides a computational ecosystem for single-cell omics data analysis. Nat Biotechnol 41, 604–606 (2023).

6. Moses, L. & Pachter, L. Museum of spatial transcriptomics. Nat Methods 19, 534–546 (2022).

7. Wolf, F. A., Angerer, P. & Theis, F. J. SCANPY: large-scale single-cell gene expression data analysis. Genome Biology 19, 15 (2018).

8. Palla, G. et al. Squidpy: a scalable framework for spatial omics analysis. Nat Methods 19, 171–178 (2022).

9. Traag, V. A., Waltman, L. & van Eck, N. J. From Louvain to Leiden: guaranteeing well-connected communities. Sci Rep 9, 5233 (2019).

10. Dong, K. & Zhang, S. Deciphering spatial domains from spatially resolved transcriptomics with an adaptive graph attention auto-encoder. Nat Commun 13, 1739 (2022).

11. Petukhov, V. et al. Cell segmentation in imaging-based spatial transcriptomics. Nat Biotechnol 40, 345–354 (2022).
