# Supplementary material for: Sopa: a technology-invariant pipeline for analyses of image-based spatial omics
Source: Nat Commun. 2024 Jun 11;15:4981. doi: 10.1038/s41467-024-48981-z (PMC11167053; doi:10.1038/s41467-024-48981-z)
Supplement: Supplementary file 2 — Reporting Summary [file 41467_2024_48981_MOESM2_ESM.pdf]

nature portfolio

Corresponding author(s): Paul-Henry Cournède

Last updated by author(s): Jan 25, 2024

Reporting Summary

Nature Portfolio wishes to improve the reproducibility of the work that we publish. This form provides structure for consistency and transparency in reporting. For further information on Nature Portfolio policies, see our Editorial Policies and the Editorial Policy Checklist.

Statistics

For all statistical analyses, confirm that the following items are present in the figure legend, table legend, main text, or Methods section.

n/a	Confirmed	
☐	☒	The exact sample size (*n*) for each experimental group/condition, given as a discrete number and unit of measurement
☒	☐	A statement on whether measurements were taken from distinct samples or whether the same sample was measured repeatedly
☒	☐	The statistical test(s) used AND whether they are one- or two-sided<br>*Only common tests should be described solely by name; describe more complex techniques in the Methods section.*
☒	☐	A description of all covariates tested
☒	☐	A description of any assumptions or corrections, such as tests of normality and adjustment for multiple comparisons
☒	☐	A full description of the statistical parameters including central tendency (e.g. means) or other basic estimates (e.g. regression coefficient) AND variation (e.g. standard deviation) or associated estimates of uncertainty (e.g. confidence intervals)
☒	☐	For null hypothesis testing, the test statistic (e.g. *F*, *t*, *r*) with confidence intervals, effect sizes, degrees of freedom and *P* value noted<br>*Give P values as exact values whenever suitable.*
☒	☐	For Bayesian analysis, information on the choice of priors and Markov chain Monte Carlo settings
☒	☐	For hierarchical and complex designs, identification of the appropriate level for tests and full reporting of outcomes
☒	☐	Estimates of effect sizes (e.g. Cohen's *d*, Pearson's *r*), indicating how they were calculated

Our web collection on statistics for biologists contains articles on many of the points above.

Software and code

Policy information about availability of computer code

Data collection	Only public datasets were used for this manuscript. Therefore, no data has been collected.
Data analysis	The code developed in this article is available as an open-source Python package called “sopa”, accessible on Github at https://github.com/gustaveroussy/sopa, and its version 1.0.0 has been used for this manuscript. The code used to run the benchmark is available at https://github.com/quentinblampey/sopa_benchmark. Finally, the version 1.3.0 of the Xenium Explorer from 10x Genomics has been used for visualization.

For manuscripts utilizing custom algorithms or software that are central to the research but not yet described in published literature, software must be made available to editors and reviewers. We strongly encourage code deposition in a community repository (e.g. GitHub). See the Nature Portfolio guidelines for submitting code & software for further information.

Data

Policy information about availability of data

All manuscripts must include a data availability statement. This statement should provide the following information, where applicable:

- Accession codes, unique identifiers, or web links for publicly available datasets
- A description of any restrictions on data availability
- For clinical datasets or third party data, please ensure that the statement adheres to our policy

The MERSCOPE dataset is freely available online at https://info.vizgen.com/merscope-ffpe-solution, and the Xenium at https://www.10xgenomics.com/resources/datasets/pancreatic-cancer-with-xenium-human-multi-tissue-and-cancer-panel-1-standard. The PhenoCycler dataset is available upon request to Akoya Biosciences,

see https://www.akoyabio.com/fusion/data-gallery/. The MACSima dataset is available upon request to Miltenyi.

Research involving human participants, their data, or biological material

Policy information about studies with human participants or human data. See also policy information about sex, gender (identity/presentation), and sexual orientation and race, ethnicity and racism.

Reporting on sex and gender	This information has not been collected, as the research is not involving human participants
Reporting on race, ethnicity, or other socially relevant groupings	This information has not been collected, as the research is not involving human participants
Population characteristics	This information has not been collected, as the research is not involving human participants
Recruitment	No patient has been recruited
Ethics oversight	There is no study protocol

Note that full information on the approval of the study protocol must also be provided in the manuscript.

Field-specific reporting

Please select the one below that is the best fit for your research. If you are not sure, read the appropriate sections before making your selection.

☒ Life sciences ☐ Behavioural & social sciences ☐ Ecological, evolutionary & environmental sciences

For a reference copy of the document with all sections, see nature.com/documents/nr-reporting-summary-flat.pdf

Life sciences study design

All studies must disclose on these points even when the disclosure is negative.

Sample size	Our manuscript is not based on a clinical study or biological trials, since we used public datasets.
Data exclusions	No data has been excluded from the analysis. For each technology, we have chosen the largest public dataset available.
Replication	The code developed is fully reproducible.
Randomization	Randomisation was not needed in the study as there were no biases from the start of the experiment. Nor did we have study groups to compare in our analysis.
Blinding	During the selection of the public datasets, we have only chosen the largest public datasets available, without first looking at the data. We used four datasets to have multiple spatial transcriptomics datasets (two, on different technologies), and several multiplex imaging datasets (two, on different technologies). This covers a significant amount of the technologies on the market.

Reporting for specific materials, systems and methods

We require information from authors about some types of materials, experimental systems and methods used in many studies. Here, indicate whether each material, system or method listed is relevant to your study. If you are not sure if a list item applies to your research, read the appropriate section before selecting a response.

Materials & experimental systems

n/a	Involved in the study	
☒	☐	Antibodies
☒	☐	Eukaryotic cell lines
☒	☐	Palaeontology and archaeology
☒	☐	Animals and other organisms
☒	☐	Clinical data
☒	☐	Dual use research of concern
☒	☐	Plants

Methods

n/a	Involved in the study	
☒	☐	ChIP-seq
☒	☐	Flow cytometry
☒	☐	MRI-based neuroimaging

Plants

Seed stocks	Not involved in the study
Novel plant genotypes	Not involved in the study
Authentication	Not involved in the study
